# 1, 9-Pyrazoloanthrones Downregulate HIF-1α and Sensitize Cancer Cells to Cetuximab-Mediated Anti-EGFR Therapy

**DOI:** 10.1371/journal.pone.0015823

**Published:** 2010-12-29

**Authors:** Yang Lu, Xinqun Li, Haiquan Lu, Zhen Fan

**Affiliations:** Department of Experimental Therapeutics, The University of Texas MD Anderson Cancer Center, Houston, Texas, United States of America; University of Bergen, Norway

## Abstract

Cetuximab, a monoclonal antibody that blocks the epidermal growth factor receptor (EGFR), is currently approved for the treatment of several types of solid tumors. We previously showed that cetuximab can inhibit hypoxia-inducible factor-1 alpha (HIF-1α) protein synthesis by inhibiting the activation of EGFR downstream signaling pathways including Erk, Akt, and mTOR. 1, 9-pyrazoloanthrone (1, 9 PA) is an anthrapyrazolone compound best known as SP600125 that specifically inhibits c-jun N-terminal kinase (JNK). Here, we report 1, 9 PA can downregulate HIF-1α independently of its inhibition of JNK. This downregulatory effect was abolished when the oxygen-dependent domain (ODD) of HIF-1α (HIF-1α-ΔODD, the domain responsible for HIF-1α degradation) was experimentally deleted or when the activity of HIF-1α prolyl hydroxylase (PHD) or the 26S proteasomal complex was inhibited, indicating that the 1, 9 PA downregulates HIF-1α by promoting PHD-dependent HIF-1α degradation. We found that the combination of 1, 9 PA and cetuximab worked synergistically to induce apoptosis in cancer cells in which cetuximab or 1, 9 PA alone had no or only weak apoptotic activity. This synergistic effect was substantially decreased in cancer cells transfected with HIF-1α-ΔODD, indicating that downregulation of HIF-1α was the mechanism of this synergistic effect. More importantly, 1, 9 PA can downregulate HIF-1α in cancer cells that are insensitive to cetuximab-induced inhibition of HIF-1α expression due to overexpression of oncogenic *Ras* (RasG12V). Our findings suggest that 1, 9 PA is a lead compound of a novel class of drugs that may be used to enhance the response of cancer cells to cetuximab through a complementary effect on the downregulation of HIF-1α.

## Introduction

The epidermal growth factor receptor (EGFR) plays several important roles in the development and progression of many types of solid tumors [1]. Over the past two decades, novel cancer therapies targeting EGFR have been developed and extensively studied [2], [3]. Recent clinical studies have demonstrated an objective response in patients with several types of cancers treated either by blocking EGFR with monoclonal antibodies (cetuximab, panitumumab, etc.) or by inhibiting EGFR tyrosine kinase activity with small-molecule inhibitors (gefitinib, erlotinib, etc.) [4]–[9]. These studies led to the regulatory approval of these EGFR-targeting agents for treating colorectal, lung, and head and neck cancers in combination with conventional chemotherapy or radiotherapy; however, despite the objective responses, the overall response rate of patients treated with EGFR-targeted therapy is low, particularly when these EGFR-targeting agents are used as monotherapies [10]–[12]. Furthermore, many patients with tumors expressing or even highly expressing EGFR may not have an optimal response to treatment with the EGFR-targeting agents [3]. For example, in patients with colorectal cancer, only 20–30% of patients had disease that responded to EGFR-blocking antibodies [4]. Among the 70–80% of patients with nonresponsive disease, 30–35% had *K-Ras* mutations, 20% had *B-Raf* and *PI3K* mutations, and the rest had other aberrations [13]. Thus, although EGFR plays important roles in tumorigenesis, cancer cells are genetically unstable and can elude the effect of EGFR-targeted therapy through several well-characterized and some not-yet-known resistance mechanisms. Much ongoing research is focused on the development of novel combinatorial therapies targeting EGFR and molecules in EGFR downstream signaling pathways in an attempt to overcome these resistance mechanisms.

We previously reported that cetuximab can markedly downregulate the high basal levels of hypoxia-inducible factor-1 alpha (HIF-1α) by inhibiting HIF-1α protein synthesis in cancer cell lines that are sensitive to EGFR inhibition [14], [15]. We showed that inhibition of HIF-1α is required, although it may not be sufficient, to mediate the response of cancer cells to EGFR-targeted therapy [14]–[17]. Knockdown of HIF-1α by RNA interference (RNAi) remarkably sensitized cancer cells with oncogenic *Ras* mutations or those with *PTEN* inactivation or deletion to cetuximab treatment [16]. In contrast, overexpression of HIF-1α in cancer cells that were originally sensitive to the treatment conferred substantial resistance to anti-EGFR therapy [16]. These findings suggest that directly targeting HIF-1α may bypass several known cetuximab-resistance mechanisms, such as mutational activation of oncogenes and inactivation of tumor-suppressor genes in the EGFR downstream pathways and/or alternative activation of these downstream pathways by other growth factor receptors. Novel combination approaches to targeting EGFR and HIF-1α may, therefore, result in an improved therapeutic response in patients.

Several strategies for targeting HIF-1α or its upstream regulators or downstream target genes have been tested in recent years [18]. Approaches to directly targeting HIF-1α function include inhibiting HIF-1α gene expression using antisense or RNA interference or inhibiting the transcriptional activity of the HIF-1α/β heterodimer by interfering with its interaction with DNA or cofactors. These approaches have been mainly tested experimentally, given that they are difficult to test clinically with currently available technology. Alternatively, the HIF-1α protein can be targeted indirectly by regulating its protein synthesis or stability using pharmacologic strategies that can be tested clinically [19].

In our effort to find novel small-molecule lead compounds that have anti-HIF-1α activity and that may be further optimized for combination with cetuximab to enhance therapeutic effects in cancer cells, we discovered that 1, 9-pyrazoloanthrone (1, 9 PA), which is an anthrapyrazolone best known as SP600125 that specifically inhibits c-Jun N-terminal kinase (JNK) [20], [21], can strongly downregulate HIF-1α in multiple cancer cell lines. In this study, we studied the relationship between 1, 9 PA's known activity of inhibiting JNK and its newly discovered activity of downregulating HIF-1α. We also explored the biochemical mechanisms through which 1, 9 PA downregulates HIF-1α. Lastly, we performed proof-of-evidence experiments to test our hypothesis that treating cancer cells with a combination of cetuximab and 1, 9 PA can enhance the antitumor activity of and sensitize cancer cells to cetuximab treatment. Our findings justify the development of new derivatives of 1, 9 PA that may be used in combination with cetuximab for cancer treatment through complementary downregulation of HIF-1α without concomitant inhibition of JNK.

## Results

### 1, 9 PA downregulates HIF-1α independent of inhibiting JNK

**Figure 1:**
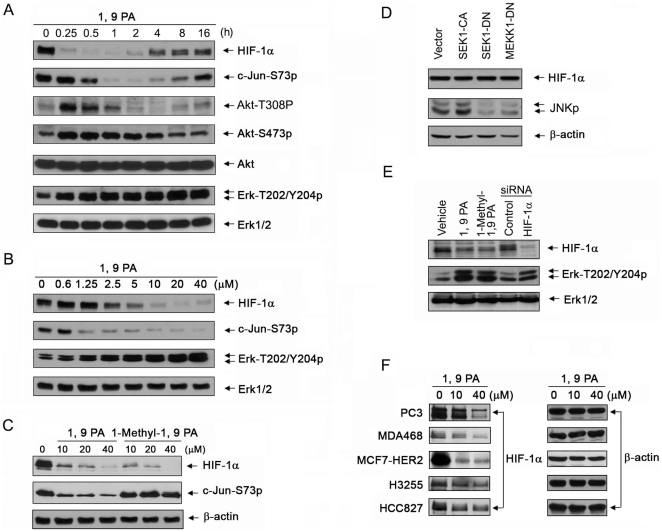
1, 9 PA downregulates HIF-1α independent of its JNK inhibitory function. (A) Temporal order and correlation of 1, 9 PA-induced HIF-1α downregulation and JNK inhibition, and the treatment-induced compensatory activation of Akt and Erk. A431 cells were treated with 5 µM 1, 9 PA in 0.5% FBS culture medium for the indicated time intervals. Cell lysates were then prepared for Western blotting with the antibodies shown. (B) Dose-dependent effects of 1, 9 PA on downregulating HIF-1α, inhibiting JNK, and activating Erk. A431 cells were exposed to increasing concentrations of 1, 9 PA for 16 h in 0.5% FBS culture medium at 37°C. Cell lysates were then prepared for Western blotting with the antibodies shown. (C) Independence of 1, 9 PA-induced HIF-1α downregulation from its JNK inhibitory function. A431 cells were treated with increasing concentrations of 1, 9 PA or 1-methyl-1, 9 PA for 1 h in 0.5% FBS culture medium. Cell lysates were then prepared for Western blotting with the antibodies shown. (D) No changes in HIF-1α level after activation or inhibition of JNK. A431 cells wert transiently transfected with a control vector or one of the constructs containing a constitutively active SEK1 S220E/T224D mutant (SEK1-CA), a dominant-negative SEK1 K129R mutant (SEK1-DN), or a dominant-negative MEKK1 K432M mutant (MEKK1-DN) overnight. Cell lysates were then prepared for Western blotting with the antibodies shown. (E) Compensatory activation of Erk after HIF-1α silencing in comparison with treatment by 1, 9 PA or 1-methyl-1, 9 PA. A431 cells were subjected to knockdown of HIF-1α with specific or control siRNA, or treated with 10 µM 1, 9 PA or 1-methyl-1, 9 PA for 16 h. Cell lysates were then prepared for Western blotting with the antibodies shown. (F) Downregulation of HIF-1α by 1, 9 PA in various cancer cell lines. Indicated cell lines were exposed to 10 or 40 µM 1, 9 PA for 1 h in 0.5% FBS culture medium. Cell lysates were then prepared for Western blotting with the antibodies shown.

In this study, we found that, in addition to inhibiting c-Jun phosphorylation in a dose-dependent manner, 1, 9 PA markedly reduced the total HIF-1α protein level in A431 vulvar squamous carcinoma cells as early as 15 min after treatment, whereas 1, 9 PA-induced inhibition of JNK was detected at a later time point (Figure 1A). This suggests that 1, 9 PA's downregulatory effect on HIF-1α is independent of its inhibitory effect on JNK. The compound also induced a transient increase in the levels of activation-specific phosphorylation of Akt on both T308 and S473, as well as a sustained and gradual increase in the level of phosphorylated Erk in the cells, which are temporally correlated with the recovery of HIF-1α level starting from approximately 4 h after treatment (Figure 1A).

The treatment-induced downregulation of HIF-1α and compensatory increase in cell signaling were also 1, 9 PA dose-dependent (Figure 1B). The minimum dose of 1, 9 PA necessary to downregulate HIF-1α was between 1.25 and 2.5 µM, which was slightly higher than the minimum dose required to effectively reduce c-Jun phosphorylation through inhibition of JNK (between 0.6 and 1.25 µM; Figure 1B), which further suggests that the newly discovered downregulatory effect of 1, 9 PA on HIF-1α is independent of its known inhibitory effect on JNK.

To confirm this observation, we treated the same cells with 1-methyl-1, 9 PA, a structural analogue of 1, 9 PA that has been used as a negative control for 1, 9 PA in literature because it does not inhibit JNK. We confirmed that 1-methyl-1, 9 PA did not reduce c-Jun phosphorylation in A431 cells compared with 1, 9 PA; however, it was equally effective at downregulating HIF-1α in these cells (Figure 1C). On the other hand, transfection of A431 cells with either a constitutively active SEK1, which activates JNK, or with dominant-negative SEK1 or dominant-negative MEKK1, both of which deactivate JNK, did not affect the level of HIF-1α in the cells (Figure 1D). These additional control experiments provided strong evidence that inhibition of JNK alone had no effect on the level of HIF-1α in the cell model examined.

Figure 1E shows that treatment of A431 cells with either 1, 9 PA or 1-methyl-1, 9 PA, or knockdown of HIF-1α expression by RNAi led to a compensatory increase in the level of phosphorylated Erk in these cells, further supporting the conclusion that the increase in cell signaling after 1, 9 PA treatment is a compensatory response that is likely related to the downregulation of HIF-1α but not to the inhibition of JNK.

In addition to A431 vulvar squamous carcinoma cells, we found that 1, 9 PA could downregulate HIF-1α in PC3 prostate cancer cells, MDA468 breast cancer cells, MCF7 breast cancer cells transfected with a high level of HER2, and H3255 and HCC827 non-small cell lung cancer cells (Figure 1F). Taken together, these findings strongly indicate that 1, 9 PA can downregulate HIF-1α in human cancer cells through a mechanism(s) that is independent of the mechanism(s) by which it inhibits JNK. These findings also suggest that cells respond to 1, 9 PA or 1-methyl-1, 9 PA-induced downregulation of HIF-1α or RNAi-mediated HIF-1α silencing through a compensatory response of activation of cell signaling pathways that are known to increase HIF-1α expression [22]–[26].

### 1, 9 PA decreases the HIF-1α level by promoting ubiquitination of HIF-1α

**Figure 2:**
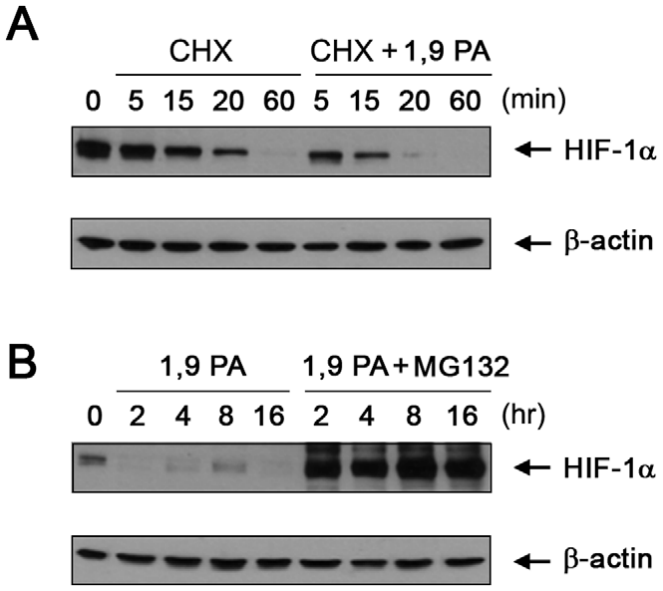
1, 9 PA downregulates HIF-1α by enhancing HIF-1α protein degradation. (A) Reduced HIF-1α protein stability in the presence of 1, 9 PA (1, 9 PA). A431 cells were treated with 10 µM cycloheximide (CHX) plus 10 µM 1, 9 PA or DMSO vehicle control in 0.5% FBS culture medium for up to 60 min at 37°C. Cell lysates were prepared after treatment at each time point and analyzed by Western blotting with the antibodies shown. (B) Inhibition of 1, 9 PA-induced HIF-1α degradation by the proteasomal inhibitor MG132. A431 cells were treated with 10 µM 1, 9 PA**±**10 µM MG132 for the indicated time intervals. Cell lysates were prepared after treatment at each time point and analyzed by Western blotting with the antibodies shown.

We then explored the mechanism(s) by which 1, 9 PA downregulates HIF-1α. HIF-1α is a highly unstable protein under normoxic conditions because it possesses the oxygen-dependent domain (ODD) that is subject to posttranslational regulation through protein ubiquitination and degradation [27], [28]. Because 1, 9 PA dramatically reduced the HIF-1α level in less than 15 min (0.25 h; Figure 1A), we hypothesized that 1, 9 PA acts through a mechanism that enhances HIF-1α protein degradation. Figure 2A shows that, compared with vehicle control treatment of A431 cells following blockade of new protein synthesis with cycloheximide, treatment of the cells with 1, 9 PA markedly expedited the degradation of HIF-1α; the degradation time was shortened from 60 min in the control cells to less than 20 min in the cells treated with 1, 9 PA. Furthermore, 1, 9 PA-induced downregulation of HIF-1α was completely blocked by treating the A431 cells with MG132 (Figure 2B), which inhibits the 26S proteasomal complex, indicating that the reduction in HIF-1α after 1, 9 PA treatment was mediated by enhancing protein degradation.

**Figure 3:**
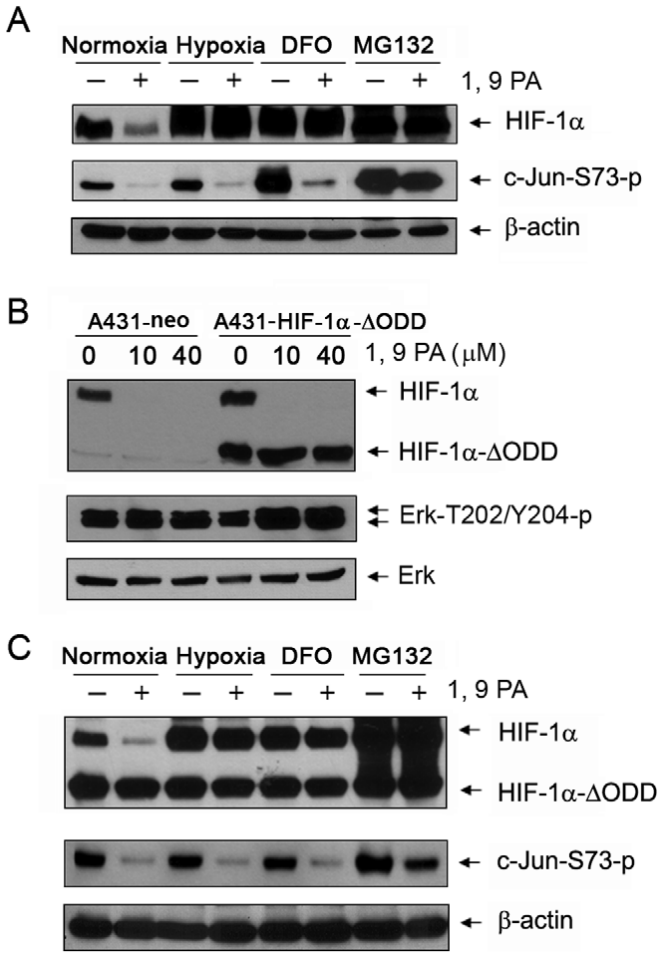
1, 9 PA downregulates HIF-1α in a PHD- and HIF-1α ODD-dependent manner. (A) Requirement of O_2_ and Fe^2+^ in the 1, 9 PA-induced downregulation of HIF-1α. A431 cells were untreated or treated with 10 µM 1, 9 PA in 0.5% FBS culture medium under normoxic conditions in the absence or presence of DFO (100 µM) or MG132 (10 µM), and under hypoxic conditions for 16 h at 37°C. Cell lysates were then prepared for Western blotting with the antibodies shown. (B) Role of the ODD of HIF-1α in the 1, 9 PA-induced downregulation of HIF-1α. A431 cells were transiently transfected with the HIF-1α-ΔODD construct or a control vector for 48 h and were then either untreated or treated with the indicated concentrations of 1, 9 PA for 1 h at 37°C. Cell lysates were then prepared for Western blotting with the antibodies shown. (C) Resistance of A431/HIF-1α-ΔODD cells to the 1, 9 PA-induced downregulation of HIF-1α. A431 cells stably expressing the HIF-1α-ΔODD construct were treated as described in (A). Cell lysates were then prepared for Western blotting with the antibodies shown.

While 1, 9 PA markedly downregulated HIF-1α under normoxic conditions, we found that the ability of 1, 9 PA to downregulate HIF-1α was markedly reduced when oxygen was not available or when the cells were treated with deferoxamine (DFO), an iron-chelating agent (Figure 3A); both O_2_ and Fe^2+^ are required for HIF-1α prolyl hydroxylase (PHD) activity to hydroxylate HIF-1α for recognition by the VHL ubiquitin ligase [29], [30]. The result of treatment with DFO was similar to that of treatment with MG132: the ubiquitinated HIF-1α was inhibited from degradation through the proteasome pathway (Figure 3A). These findings suggest that 1, 9 PA can directly or indirectly affect the ubiquitination of HIF-1α [29], [30].

To further test this hypothesis, we examined the ability of 1, 9 PA to regulate an HIF-1α mutant in which the PHD/VHL interacting domain on HIF-1α, i.e., ODD, was deleted (HIF-1α-ΔODD). As expected, while 1, 9 PA decreased the level of wild-type HIF-1α, it did not affect the level of HIF-1α-ΔODD in A431 cells transiently transfected with the HIF-1α-ΔODD construct (Figure 3B). We then established pooled A431 cells that stably expressed HIF-1α-ΔODD and further confirmed the findings obtained in the parental A431 cells by treating the A431 cells expressing HIF-1α-ΔODD in the same manner (Figure 3C). Together, these findings strongly support the conclusion that 1, 9 PA downregulates HIF-1α through a mechanism involving the interaction between PHD/VHL and the ODD of HIF-1α. When the key elements (O_2_, Fe^2+^, and 26S proteasome) required for PHD activity to hydroxylate HIF-1α and for the subsequent VHL-mediated ubiquitination and degradation are missing or inhibited, or when the ODD of HIF-1α, which is the targeted domain by the VHL ubiquitin ligase complex, is absent, 1, 9 PA loses its ability to downregulate HIF-1α.

**Figure 4:**
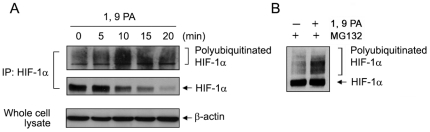
1, 9 PA enhances HIF-1α ubiquitination. (A). A431 cells were treated with 10 µM 1, 9 PA for indicated time period. Cell lysates were prepared for HIF-1α immunoprecipitation, followed by Western blotting of the immunoprecipitates with antibodies directed against ubiquitin (top), HIF-1α, and β-actin. (B) A431 cells were untreated or treated with 10 µM 1, 9 PA in the presence of 10 µM MG132 for 1 h in 0.5% FBS medium. HIF-1α was immunoprecipitated followed by Western blot analysis with an anti-HIF-1α antibody.

To obtain direct evidence that 1, 9 PA promotes the ubiquitination of HIF-1α, we subjected the HIF-1α immunoprecipitated from lysates of 1, 9 PA-treated A431 cells to Western blotting with an anti-ubiquitin antibody. We found that the level of polyubiquitinated HIF-1α was clearly increased within 10 minutes after 1, 9 PA treatment (Figure 4A). Presence of the proteasomal inhibitor MG132 during the 1, 9 PA treatment inhibited ubiquitinated HIF-1α from being degraded (Figure 4B).

Taken together, our data provide strong evidence that 1, 9 PA decreases HIF-1α through a mechanism that enhances the interaction between the ODD of HIF-1α and the PHD/VHL/ubiquitination complex, and thereby leads to an enhanced degradation of HIF-1α through the proteasome pathway.

### 1, 9 PA synergizes with cetuximab to induce apoptosis through co-downregulating HIF-1α

**Figure 5:**
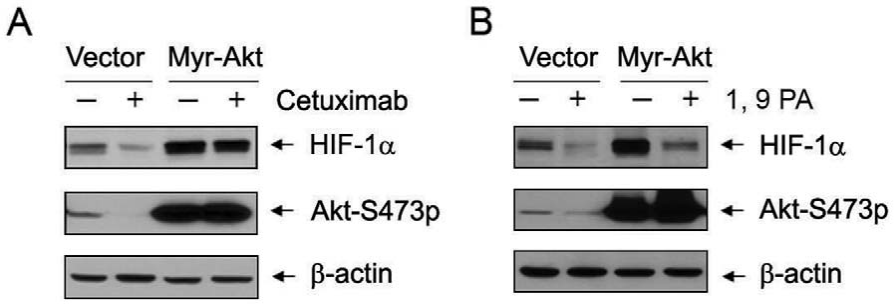
Differential effect of constitutively active Akt on cetuximab and 1, 9 PA-induced downregulation of HIF-1α. A431 cells were transiently transfected with a control vector or a myristoylated Akt (Myr-Akt) for 24 h in 0.5% FBS medium. The vector- or Myr-Akt–transfected cells were then treated with either 20 nM cetuximab or PBS overnight (A), or with 10 µM 1, 9 PA or DMSO vehicle control for 1 h (B). After treatment, cell lysates were prepared for Western blotting with the antibodies shown.

We previously showed that cetuximab downregulates HIF-1α in cancer cells sensitive to anti-EGFR therapy through inhibition of HIF-1α protein synthesis, which was prevented by expression of a myristoylated Akt that is constitutively active [16]. Figure 5A shows our current findings, which are similar to those we previously reported; interestingly, unlike our previous findings, expression of the constitutively active Akt did not affect 1, 9 PA-induced downregulation of HIF-1α (Figure 5B), implicating that the role of 1, 9 PA-induced increase in Akt activity (see Figure 1A) was not to counteract 1, 9 PA-induced HIF-1α protein degradation, but rather to compensate for 1, 9 PA-induced HIF-1α protein degradation by stimulating new HIF-1α protein synthesis.

**Figure 6:**
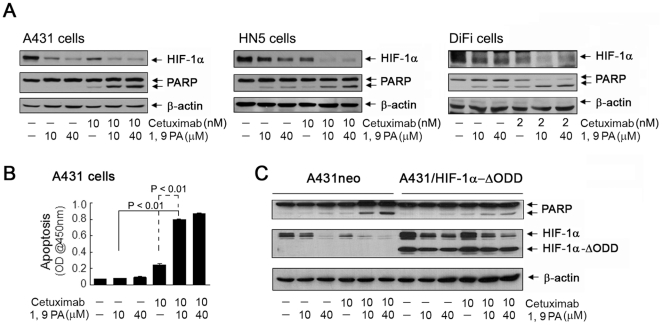
Combination of 1, 9 PA and cetuximab induces apoptosis through downregulation of HIF-1α. (A) Induction of PARP cleavage by the combination of 1, 9 PA and cetuximab. A431, HN5, and DiFi cells were untreated or treated with cetuximab (10 nM for A431 and HN5 cells and 2 nM for DiFi cells for 16 h), 1, 9 PA (10 µM or 40 µM added the last hour before cell lysis), or both in 0.5% FBS culture medium. Cell lysates were prepared and analyzed by Western blotting with the antibodies shown. (B) Increased induction of apoptosis by the combination of 1, 9 PA and cetuximab. A431 cells were treated as described in (A). Cell lysates were prepared and analyzed by apoptosis ELISA. The relative absorbance values are plotted. The *p* value was <0.01 when comparing the level of apoptosis by 1, 9 PA alone (10 or 40 µM) or cetuximab alone with that of apoptosis by combination of the 2 agents (note: only the *p* values comparing 10 µM 1, 9 PA alone and in combination with cetuximab are shown). (C) Dependence of induction of apoptosis by the combination of 1, 9 PA and cetuximab on HIF-1α downregulation. A431neo and A431/HIF-1α-ΔODD cells were treated as indicated, and the cell lysates were prepared and analyzed as described in (A).

We thus hypothesized that the combination of 1, 9 PA and cetuximab, which can inhibit the Akt and Erk pathways and thereby block the compensatory mechanisms, would have an additive or even synergistic effect on the downregulation of HIF-1α. We tested this hypothesis in 3 cancer cell lines that are sensitive to cetuximab treatment: A431, HN5 (head and neck cancer), and DiFi (colorectal cancer). Because DiFi cells are extremely sensitive to cetuximab, they were treated with 2 nM cetuximab; A431 and HN5 cells, which are not as sensitive to cetuximab as DiFi cells, were treated with 10 nM cetuximab. Figure 6A shows that, when any of the 3 cell lines was treated with either 10 or 40 µM 1, 9 PA or cetuximab alone, HIF-1α was downregulated. When the cells were treated with both 1, 9 PA and cetuximab, HIF-1α was further downregulated. Treating the cells with cetuximab or 1, 9 PA alone induced only minimal or no cleavage of PARP, a marker of apoptosis; however, the combination of cetuximab and 1, 9 PA markedly enhanced the induction of PARP cleavage in all 3 cell lines.

We selected A431 cells to further confirm this result by using an independent apoptosis ELISA that quantitatively measures the level of histone-associated DNA fragmentation in the cytoplasm after apoptosis (Figure 6B). 1, 9 PA alone at concentrations of 10 and 40 µM did not noticeably increase the level of apoptosis; cetuximab alone only moderately increased the level of histone-associated DNA fragmentation in the cytoplasm. However, we found that the combination treatment significantly increased the level of apoptosis (*p*<0.01). To further confirm whether the induction of apoptosis seen with the combination treatment was mediated by the complementary effects on the downregulation of HIF-1α by these 2 agents, we repeated the experiments in A431 cells expressing HIF-1α-ΔODD cells. Figure 6C shows that, compared with its effect in control vector–transfected A431neo cells, the combination treatment had a markedly lower effect on the PARP cleavage in A431/HIF-1α-ΔODD cells. The effect of 1, 9 PA on the downregulation of endogenous HIF-1α was also reduced in A431/HIF-1α-ΔODD cells compared with A431neo cells. These data indicate that downregulation of HIF-1α was the mechanism for the synergic effect of the combination of 1, 9 PA and cetuximab.

### 1, 9 PA overcomes oncogenic Ras-induced cetuximab resistance

**Figure 7:**
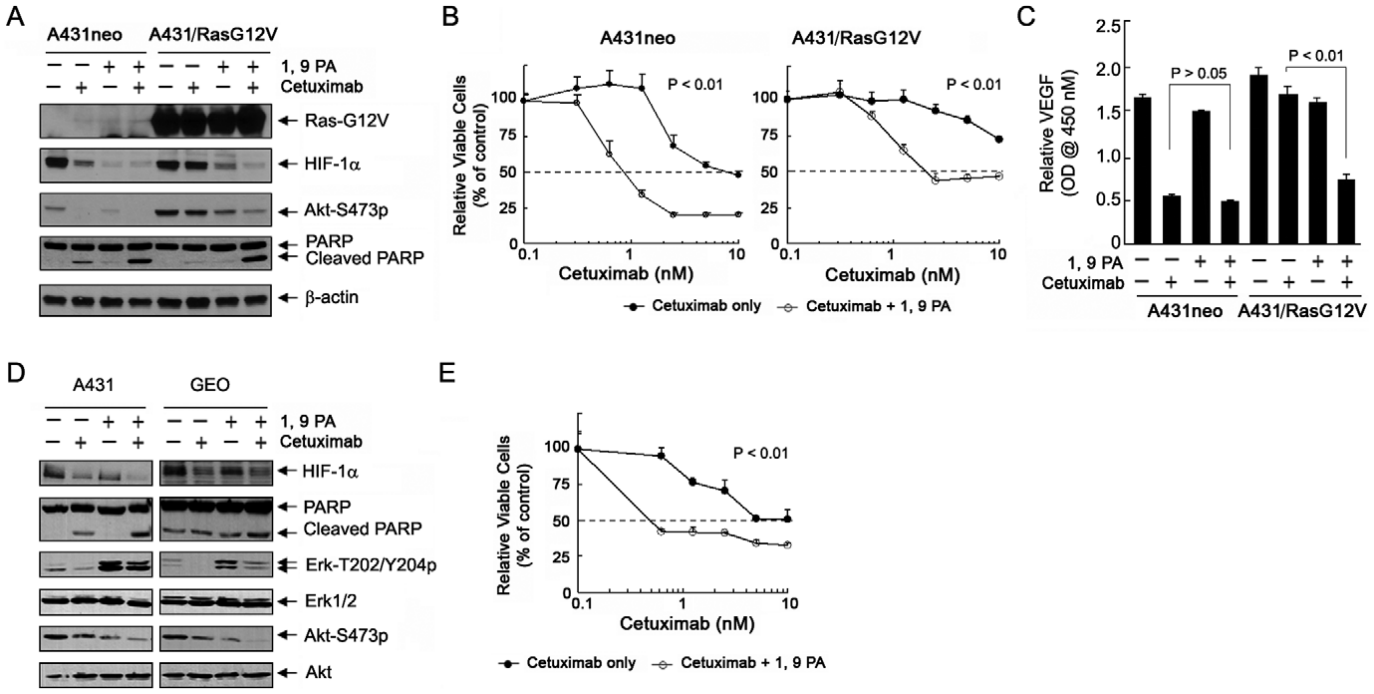
1, 9 PA enhances responses of cancer cells expressing an oncogenic Ras mutant to cetuximab. (A) Effect of 1, 9 PA and cetuximab, either alone or in combination, on the HIF-1α level and induction of apoptosis. A431neo and A431/RasG12V cells were untreated or treated with cetuximab (10 nM for 16 h), 10 µM 1, 9 PA (added the last hour before cell lysis), or both in 0.5% FBS culture medium. Cell lysates were prepared and analyzed by Western blotting with the antibodies shown. (B) 1, 9 PA-mediated sensitization to cetuximab-induced growth inhibition. A431neo and A431/RasG12V cells were treated with increasing concentrations of cetuximab ±5 µM 1, 9 PA in 0.5% FBS culture medium for 5 days. After treatment, the cells were subjected to an MTT assay. The optical density values of the treated groups were normalized to the values of the control groups (with or without 1, 9 PA treatment) and expressed as a percentage of respective control. The percentage of surviving cells was plotted as a function of treatment with increasing concentrations of cetuximab. The differences in cell survival between the two groups were statistically significant (*p*<0.01) when the concentrations of cetuximab were greater than 0.625 nM in A431neo cells and 1.25 nM in A431/RasG12V cells. (C) 1, 9 PA-mediated sensitization to cetuximab-induced inhibition of VEGF production. A431neo and A431/RasG12V cells were untreated or treated with 10 nM cetuximab, 10 µM 1, 9 PA, or both in 0.5% FBS culture medium for 16 h. The VEGF secreted into the conditioned media by the cells was measured by ELISA. The *p*-values for indicated comparisons were shown. (D) Comparison of A431 and GEO cells to treatment with 1, 9 PA and cetuximab, either alone or in combination. A431 and GEO cells were treated as described in (A). Cell lysates were prepared and analyzed by Western blotting with the antibodies shown. (E). 1, 9 PA-mediated sensitization GEO cells to cetuximab-induced growth inhibition. GEO cells were treated with increasing concentrations of cetuximab ±5 µM 1, 9 PA in 0.5% FBS culture medium for 5 days. After treatment, the cells were subjected to an MTT assay. The data were processed as described in (B). The differences in cell survival between the two groups were statistically significant (*p*<0.01) at all concentrations of cetuximab tested.

Oncogenic mutation of *Ras* has been shown to be a major mechanism of cetuximab resistance in patients with colorectal cancer [10]. We previously showed as a proof of concept that knockdown of HIF-1α through RNAi substantially restores the sensitivity of A431 cells transfected with an oncogenic *H-Ras* (G12V) mutant to cetuximab treatment [16]. To further prove this novel concept, we examined the effect of 1, 9 PA alone and in combination with cetuximab in A431 cells transfected with *Ras-G12V* (A431/RasG12V). Figure 7A shows that, compared with control vector–transfected A431neo cells, A431/RasG12V cells had a higher basal level of Akt phosphorylation and were resistant to cetuximab-induced inhibition of Akt phosphorylation, downregulation of HIF-1α, and cleavage of PARP. In addition, 1, 9 PA alone inhibited both the basal and Ras-induced upregulation of Akt phosphorylation and downregulated HIF-1α in both A431neo and A431/RasG12V cells, but it had no detectable effect on the induction of PARP cleavage in either cell line. The combination of 1, 9 PA and cetuximab, however, markedly increased cleavage of PARP in both A431neo and A431/RasG12V cells. The cleavage of PARP in A431/RasG12V cells is particularly important, because cetuximab alone was unable to induce PARP cleavage in these cells owing to the expression of oncogenic *Ras*; whereas the combination treatment in the A431/RasG12V cells resulted in a level of PARP cleavage that was similar to the level of PARP cleavage in A431neo cells, indicating a synergism between 1, 9 PA and cetuximab in inducing apoptosis in cetuximab-resistant cancer cells.

To obtain further evidence at a cellular level to support the pro-apoptotic effect of the combination treatment, we performed a conventional cell growth and survival assay comparing the cetuximab dose-dependent effect with and without 1, 9 PA in A431neo and A431/RasG12V cells. We found that 1, 9 PA sensitized both A431neo and A431/RasG12V cells to cetuximab (Figure 7B). The addition of 1, 9 PA to cetuximab shifted the IC_50_ of cetuximab from 3–10 nM to less than 1 nM in A431neo cells, and more importantly, it achieved an IC_50_ of 3 nM for cetuximab in A431/RasG12V cells; in contrast, the IC_50_ of cetuximab alone was not reached (Figure 7B), even at concentrations greater than 10 nM (data not shown).

We found that treating A431neo cells with 1, 9 PA alone did not appreciably inhibit VEGF production compared to treating these cells with cetuximab alone; adding 1, 9 PA did not further lower the level of VEGF production that was inhibited by cetuximab (Figure 7C). Interestingly, however, when A431 cells became resistance to cetuximab-induced inhibition of VEGF production due to expression of the oncogenic RasG12V, the addition of 1, 9 PA significantly lowered the level of VEGF production (*p*<0.01).

Lastly, to determine whether 1, 9 PA can also sensitize cancer cells with naturally occurring *Ras* mutations, we examined the effect of the combination of 1, 9 PA and cetuximab on GEO colorectal cancer cells, which are known to have mutated *Ras* [31]. However, despite bearing a mutated *Ras* on exon 2, GEO cells responded to 1, 9 PA and cetuximab in a similar fashion as A431 cells did. 1, 9 PA led to a compensatory increase in the level of phosphorylated Erk after overnight treatment (16 h) in both A431 and GEO cells, which was reduced in the presence of cetuximab. The combination of these 2 agents enhanced PARP cleavage in both types of cells (Figure 7D) and sensitized GEO cells to cetuximab-induced inhibition of cell growth and survival (Figure 7E).

In summary, our findings indicate that 1, 9 PA can sensitize cancer cells to cetuximab-mediated anti-EGFR therapy by downregulating HIF-1α and can enhance cellular response to cetuximab treatment in cancer cells bearing oncogenic *Ras* mutations.

## Discussion

Here, we report 2 important findings, which, to our knowledge, have not been previously reported. First, we found that 1, 9 PA promotes HIF-1α ubiquitination and degradation, and this function of 1, 9 PA is independent of its well-known function as an inhibitor of JNK. Second, we found that 1, 9 PA sensitizes cancer cells to cetuximab treatment, and this effect of 1, 9 PA is dependent on the ability of 1, 9 PA to downregulate HIF-1α. Furthermore, we showed that 1, 9 PA can enhance the response of cancer cells expressing an oncogenic *Ras*, which is a well-characterized cetuximab-resistance mechanism in colorectal cancer patients, to cetuximab [10]–[12], [32]–[37]. Thus, our work establishes an important principle for the first time that 1, 9 PA, as a lead compound, can sensitize cancer cells to cetuximab-mediated anti-EGFR therapy. Development of new derivatives of 1, 9 PA that will retain the activity of downregulating HIF-1α but will not affect its inhibitory effect on JNK activity is warranted.

Our studies showed that 1, 9 PA downregulates HIF-1α by enhancing HIF-1α degradation; however, the exact molecular target for this novel function of 1, 9 PA remains unknown. Anthrapyrazolones as a class are capable of interacting with DNA, which may affect HIF-1α mRNA stability or synthesis; however, such an interaction with DNA is not supposedly dependent on PHD and the structural requirement of the ODD of HIF-1α. Because 1, 9 PA apparently accelerates degradation of HIF-1α by enhancing HIF-1α ubiquitination, one possibility is that 1, 9 PA activates a pathway that enhances ubiquitination of HIF-1α. This could be mediated by the increased expression and/or activity of related proteins, such as PHD, the VHL ubiquitin ligase, a protein that activates PHD or VHL, or a protein that deactivates an inhibitor of VHL or PHD. Other possible mechanisms by which 1, 9 PA promotes the degradation of HIF-1α are targeting other proteins indirectly affecting HIF-1α degradation through the PHD/VHL ubiquitination pathway. Further investigations on identifying the molecular target(s), interaction with which 1, 9 PA promotes HIF-1α ubiquitination, are needed to develop this lead compound into a new class of anticancer agents.

Our current work showing that the combination of 1, 9 PA and cetuximab induces apoptosis in various cancer cells has strong implications for clinical application. There are 2 important potential concerns that are worth discussing. First, we found that there was quick activation of compensatory cell signaling after 1, 9 PA treatment, which can stimulate new HIF-1α protein synthesis and quickly revert the lowered HIF-1α level to its original level before treatment. Second, 1, 9 PA-mediated HIF-1α ubiquitination requires the presence of oxygen but most solid tumors are hypoxic. These 2 potential problems can be solved by our proposed strategy of combining 1, 9 PA and cetuximab. Here, we demonstrated that cetuximab can inhibit the cell signaling activated in the cells as a compensatory response after 1, 9 PA treatment; the combination treatment resulted in synergistic effects on induction of cell death via apoptosis. In addition, although hypoxia is common in most solid tumors, tumors are not 100% hypoxic. Some tumor areas, particularly those located at the periphery of a solid tumor, are not hypoxic. Importantly, cetuximab can inhibit HIF-1α protein synthesis in both normoxic and hypoxic cells [16]. Together, these considerations strongly justify the combination of cetuximab with new derivatives of 1, 9 PA in future preclinical (animal studies) and clinical studies.

Exploration of new strategies combining EGFR inhibitors with agents targeting one or more EGFR downstream targets, such as signal transduction molecules in the PI3K/Akt/mTOR pathway, has been an active area of research in recent years [38]. Based on our current and recent findings [14]–[17], we propose that an alternative co-targeting of EGFR with one or more of the critically important downstream effector molecules, such as HIF-1α, may be a better approach. The advantage of directly targeting critical transcription factors is that it bypasses several intermediate signal transduction molecules in the EGFR downstream signaling pathways that are often aberrantly regulated by mutations in cancer cells. The complementarity between the mechanisms used by cetuximab (inhibition of HIF-1α protein synthesis) and 1, 9 PA (promotion of HIF-1α protein degradation) for downregulating HIF-1α offers a strong rationale for a new combination to effectively inhibit the HIF-1 transcription factor-mediated cellular effects. The results shown in our current study clearly demonstrate a strong induction of apoptosis in cancer cells by combination treatment using cetuximab and 1, 9 PA that acted through targeting HIF-1α. Further investigation in proper animal models with new derivatives of 1, 9 PA is needed to provide further evidence supporting this novel strategy.

In summary, we have identified a novel activity of 1, 9 PA—downregulation of HIF-1α—that is independent of its JNK activity, and we have elucidated the relevant mechanism of action. We have also explored potential application of this novel activity of 1, 9 PA in enhancing cancer cell response to cetuximab. Our findings provide a strong rationale for developing new derivatives of this lead compound that could be used in combination with cetuximab in cancer patients to improve clinical outcomes.

## Materials and Methods

### Reagents

Cetuximab was a gift from ImClone Systems (New York, NY). 1, 9 PA, 1-methy-1, 9 PA, and MG132 were purchased from CalBiochem/EMD Chemicals, Inc. (Gibbstown, NJ). The following antibodies were used for Western blotting and immunoprecipitation: HIF-1α and Ras (BD Biosciences Pharmingen, San Diego, CA); total and S473-phosphorylated Akt, total extracellular signal-activated kinase (Erk), total and S73-phosphorylated c-Jun, poly(ADP-ribose) polymerase (PARP), and ubiquitin (all from Cell Signaling Technology, Inc., Danvers, MA); T202/Y204-phosphorylated Erk (Santa Cruz Biotechnology, Santa Cruz, CA). All other chemicals were purchased from Sigma-Aldrich Corp. (St. Louis, MO).

### Cell lines and cell culture

A431 vulvar squamous carcinoma cells, HN5 head and neck cancer cells, PC3 prostate cancer cells, MDA468 and MCF7-HER2 breast cancer cells, and H3255 and H827 non-small cell lung cancer cells, DiFi and GEO colorectal cancer cells were obtained and maintained as described previously [15], [16], [39]–[41]. Briefly, all cell lines were grown in Dulbecco's modified Eagle's medium and Ham's F12 medium (50∶50 by volume) supplemented with 10% fetal bovine serum (FBS), 2 mM glutamine, 100 U/mL of penicillin, and 100 µg/mL of streptomycin and maintained in a humidified 37°C incubator with 95% air and 5% CO_2_. For hypoxic cell cultures, cells were placed in an airtight chamber that was flushed with a gas mixture of 5% CO_2_ and 95% N_2_. The oxygen concentration inside the chamber was maintained at 1% using the Pro-Ox O2 regulator (Model 110; BioSpherix, Lacona, NY). The hypoxic chamber was placed in the same 37°C incubator where parallel groups of cells were incubated under normoxic culture conditions.

### cDNA constructs and transfection

cDNA constructs expressing HIF-1α-ΔODD, Myr-Akt, and H-RasG12V were generated as previously described [16], [42]. Constructs expressing constitutively active SEK1 S220E/T224D mutant (SEK1-CA), dominant-negative SEK1 K129R mutant (SEK1-DN), and dominant-negative MEKK1 K432M mutant (MEKK1-DN) were kindly provided by Dr. Jonathan Kurie (MD Anderson Cancer Center). Transfection of the cDNA constructs into the targeted cells was performed with Lipofectamine 2000 (Invitrogen, Inc. Carlsbad, CA), according to the manufacturer's instructions.

### Western blotting

Cultured cells were harvested with a rubber scraper and washed twice with cold phosphate-buffered saline (PBS). Cell pellets were lysed and kept on ice for at least 10 min in a buffer containing 50 mM Tris (pH 7.4), 150 mM NaCl, 0.5% Nonidet P-40, 50 mM NaF, 1 mM Na_3_VO_4_, 1 mM phenylmethylsulfonyl fluoride, 25 µg/mL of leupeptin, and 25 µg/mL of aprotinin. The lysates were cleared by centrifugation, and the supernatants were collected. Cell lysates were then separated by sodium dodecyl sulfate (SDS)-polyacrylamide gel electrophoresis and subjected to Western blotting with the primary antibodies and horseradish peroxidase–labeled secondary antibodies. We visualized the signals with an enhanced chemiluminescence detection kit (GE Healthcare, Piscataway, NJ).

### Cell proliferation assay

Cells were cultured in 24-well plates with 0.5 mL of medium per well at 37°C in a CO_2_ incubator. At the end of the desired treatment in cell culture, the cells were incubated for an additional 2 h after the addition of 50 µL/well of 10 mg/mL 3-(4,5-dimethylthiazol-2-yl)-2,5-diphenyltetrazolium bromide (MTT). The cells were then lysed with a lysis buffer (500 µL/well) containing 20% SDS in dimethyl formamide/H_2_O (1∶1, v/v; pH 4.7) at 37°C for at least 6 h. We determined the relative number of surviving cells in each group by measuring the optical density (OD) of the cell lysates at an absorbance wavelength of 570 nm. The OD value in each treatment group was then normalized to that of untreated cells as a percentage of the OD value of the control and plotted against the treatments.

### Apoptosis assay

We measured apoptosis using an enzyme-linked immunosorbent assay (ELISA) kit (Roche Diagnostics Corp., Indianapolis, IN) that quantitatively measures cytoplasmic histone-associated DNA fragments (mononucleosomes and oligonucleosomes) and by Western blotting with an antibody that recognizes both uncleaved and cleaved PARP after various treatments, as previously described [43], [44].

### Measurement of VEGF levels in conditioned medium

VEGF levels in the conditioned medium were measured by ELISA (R & D Systems, Minneapolis, MN) 24 h after treatment. Relative VEGF levels were expressed as the OD value of the conditioned medium normalized by the number of cells of each sample in the culture plates. Experiments were repeated twice.

### Statistical analysis

Student's t-test was used to compare the mean differences between two groups using Statistica-6.0 software package. The results were expressed as means ± standard deviation. P values<0.01 were considered statistically significant.
